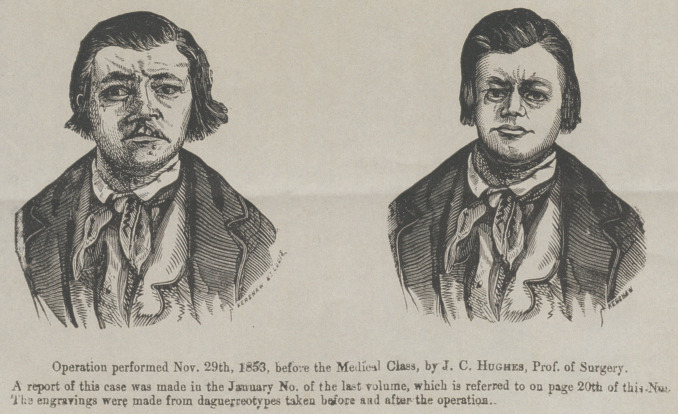# Notes on Anatomy

**Published:** 1854-11

**Authors:** J. C. Hughes

**Affiliations:** Professor of Surgery in the Medical Department of the Iowa University


### IOWA MEDICAL JOURNAL.

VOL. II. KEOKUK, IOWA, OCT. & NOV., 1854. NO. I.

### ORIGINAL COMMUNICATIONS.

### NOTES ON ANATOMY.

BY J. C. HUGHES, M. D.,

Professor of Surgery in the Medical Department of the Iowa University.

(Reported to the State Medical Society, 1854.)

As Chairman of a Committee appointed by this Association, upon one of the most important branches of the Science, *that of Anatomy*, would submit to your honorable body the following Report:

Before proceeding to notice any particular investigations, I shall be pardoned no doubt, for offering a few thoughts upon that branch of study which is regarded by every tyro, about to enter upon its investigation, and indeed by too many who have already prosecuted it, as *dry*, *difficult*, and uninteresting. This serious error should be combated on every occasion, and its unfortunate consequences exhibited as a warning incentive to greater zeal in the cultivation of anatomy. To the absence of a competent knowledge of anatomy in the earlier ages, was attributable the great want of surgical skill, and the inefficiency of this department so important to human life.

The details of Anatomy are not *dull*, or dry; on the contrary, no subject can be more interesting than the enquiry into the structure of organized bodies. The structure of the earth, its mountains, hills, and plains, are subjects of the liveliest interest, and have engaged and are now engaging the attention and arduous investigation of the most learned and scientific of the past and present day; but how much more interesting is that enquiry which seeks to learn the structure of the human body.

The practical benefits which result to the human family from a knowledge of the structure of the inorganic matter composing the earth's structure, sink into insignificance when compared to those advantages derived from a knowledge of the structural formation of the human body. The physiologist in his discussions upon the laws of life, discourses eloquently of the relations subsisting between different organs, and the offices performed by each; but, after all, he owes to the anatomist his knowledge of the minute structure by which this office was performed, and all his rich and beautiful elucidations of function are gleaned from and are the results of the practical, patient, and laborious researches of the anatomist.

What would he know of the secretion of *bile* by the liver, unless minute investigations had disclosed numerous primary cells? What would we know of the *aeration* of the blood, did not the anatomist discover the air vesicles capped over by a tissue or network of vessels carrying blood, seeking to be vitalized? He might, by the aid of the microscope, detect the spermatozoa in the seminal secretion, but what would he know of fecundation and conception had not the knife exposed the ovaries, the Graafian vesicles and the canal of the Fallopian tubes, terminating in the uterus. The truth is, physiology could not exist without anatomy, and it never did until anatomy was cultivated. And yet, Anatomy in the abstract would prove but little more than a map or the description of a complicated machine which in its parts could be separately described and the relations given; but it required physiology to describe the action and uses of each part, and their action and uses in the aggregate, in a normal state and condition.

An examination of the structure would necessarily result in a just comprehension of its actions and purposes, for it would be very singular indeed if the mind were to cease its enquiries simply upon an examination of its structural formation. Because Anatomy formed the basis of physiology, it follows that in proportion as the former advances, the latter will improve, hence the necessity for its further and more thorough cultivation.

Anatomy and Physiology explain the normal condition of the parts in health. They fix the standard, which is of vital importance, and because of this they constitute the foundation of the Science of Medicine.

When the medical man passes forth to the performance of his duty, armed and prepared to contend against disease, he must be familiar with healthy structure, in order that he may be able to institute profitable comparisons between this and a diseased condition of parts, or in other words, he must know what is normal before he can certainly decide upon the abnormal.

Not only is he required to know the abnormal condition of the *structures* of organs, but he must know also the abnormal condition of *function*, the dependence of one organ upon another, and the relations subsisting between each.

These relations take place through nervous and vascular arrangements and connexions, and here again the physiologist appeals to anatomy for the facts as revealed by the scalpel. An analysis leads him to the adoption of the doctrines of reflex functions, reflected irritation and sensation through the agency of the nervous centres and the sensory nerves.

The Pathologist follows closely in his path and finds excessive pain in the sacral region and calling upon the anatomist, the nervous arrangement is explained, and the physiologist suggests irritation at the sentient extremities of the sacral nerves, and in the progress of his diagnosis he will most likely find the true seat of disease in the rectum. Further on in the progress of duty he is required to examine a painful knee, but all the other symptoms of iuflammation are wanting, and the anatomist is now required to explain the nervous arrangement here, and the physiologist describes the *modes operandi* of reflected sensation, and he readily diagnoses the disease to be coxalgia; or he finds the spleen enormously engorged, and his anatomy describes this organ as a vascular gland and capable of great distention, and also that there is a vascular connexion with the liver. The Physiologist explains the supposed office of the spleen in sharing the congestion with the liver, and the diagnosis will be that of portal congestion. But it is not necessary here to multiply instances.

The subject of Anatomy acquires increased importance when considered in its relation to Surgery. It were needless in me to say that it is indispensable. Without it the operator cannot make one single movement unless he thereby incurs the greatest risk to the life of the patient. In all the capital operations whether of Lithotomy, Herniotomy, or any of the other feats in Surgery, without understanding the structures which he invades with the knife, he will be in danger of Scylla on the one hand, and Charybdis on the other.

He may be called upon to check an alarming hemorrhage which threatens the life of his patient. If he knows nothing of the location of the vessel whence it flows, he will fail of his duty, and be compelled to look upon the vital spark as it flickers upon the altar, and to see his patient sink into the grave. Methinks I see his tremulous lip whisper—"would I were an Anatomist."

After these few remarks, I shall proceed to speak of some variations from the regular order of distribution of the muscles, arteries, &c., as described by our different authors, as well as refer to one or two muscles which I have not seen mentioned or described by any author.

In the winter of 1852 and '53, while preparing my dissections for demonstration before the class, I found in dissecting the Auricular group of muscles, a beautiful little muscle from 20 to 24 lines in length, and from 4 to 6 lines in width, situated about half an inch below the *posterior auris*, arising from the fascia which bound down the *Sterno-Cleido mastoid* muscle, and also from the mastoid portion of the temporal bone. Tracing the muscle carefully, I found it to be inserted into the posterior portion of the concha opposite the *meatus auditorius externus*, and seemed to be intended as a dilator of that opening. It had no connection with the proper muscles of that region the *Retrahens auris*, which is sometimes found to have one or more fleshy fasciculi. We will now leave the Auricular group and pass to that group known as the elevators of the Os Hyoides and Larynx. From the anterior belly of the *Digastricus* muscle, I have found in nearly all the subjects I have dissected a distinct set of fibres arising from the *Supra-Hyoidean* fascia which has not been described by any Anatomist, unless Cruveilhier when he speaks of a small fasciculus arising from the Os Hyoides and strengthening the anterior belly of that muscle. The fibres seem to belong to that muscle, but by many are separated, supposing them to be a distinct muscle, since the direction of fibres in connection with the fascia, does not correspond with the direction of the fibres of the anterior belly of the muscle.

In the dissection of the superficial group of the neck by one of the students, I was called to notice the origin of the *Sterno-Cleido Mastoid.* It arose by a broad belt from the Clavicle, no portion of it originating from the sternum, nor did it present any of that separation which has been regarded as always present.

In the Thoracic group, the *Pectoralis Minor* was not only inserted into the Corocoid process of the Scapula, but had its tendonous fibres mingled with the *Coroco, Brachialis*, and with the short head of the *biceps*, for the distance of more than an inch, the connection being very firm.

In the anterior and posteror scapular regions, I found the *subscapularis* to be inserted not only into the lesser tuberosity of the humerus, but also into the capsule of the joint, and the *supra-spinatus*, *infra spinatus* and *teres minor* inserted into the same capsule as well as into the greater tuberosity.

Passing down the arm, we come to the *pronators* and *flexors*, having their origins principally from the inner cordyle of the humerus. There is no student, or I might say practical anatomist, who is not confused when he attempts to trace out their origin as described. And the profession are, and well might be, satisfied with knowing the object of each, without their exact points of beginning. Coming to the *Abductor pollicis* which by most anatomists is spoken of as a very small muscle, we find upon examination that it is about two inches in length and one in width, covering in nearly the whole of the *Flexor brevis pollicis* and *Flexor ossi meticarpi.* I have frequently separated this muscle, supposing I had two abductors when in fact there was but one. Again, the *abductor minimi digiti* is represented as being a very small muscle, when in truth looking to the position it occupies, it is of considerable size, and not only arises from the pisiforme bone, but often has a distinct fleshy fasciculus from the annular ligament.

The *lumbricali* muscles, four in number, arose as described excepting the two which should have arisen from the tendons of the *flexor profundus.* In this instance both arose from the tendon which passed to the little finger, the insertion between the ring and middle fingers was by two tendons which is sometimes the case.

The *Serratus magnus* of the lateral thoracic region, instead of its natural origin, arose from the eight superior ribs. The first part from the first and second, and the second part from the second and third. The fibres of the second part commencing anterior to those of the first; the third part arising by six fleshy digitations from the third, fourth, fifth, sixth, seventh and eighth ribs. These digitations were very distinct, five of them indigitating with the external oblique. The insertion of the three parts correspond with Wilson's description, but not their origin.

In the Gluteal region, the *quadratus femoris* was unusually small, not over half an inch in width. The *obturator externus* very large. The *vastus internus* and *externus* had their origins, not only from the edge of the patella, but also from the tendon of the *rectus* for a distance of some three inches.

The *Illiacus* of the internal femoral region arose from the lower border of the anterior inferior spinous process of the ilium; also, from the outer surface of that region and from the capsule of the joint, as well as the inner concave surface of the ilium. The insertion of the common tendon of the *Illiacus* and *Psoas* into the lesser *trochanter* was as usual, but in addition to this there was a broad insertion from one and a half to two inches, seeming to belong properly to the *illiacus*, which was inserted into the line leading from the anterior inter trochanteric line to the linea aspera.

The *Pectineus* arose not only from the pectineal line, but from the surface of bone in front, also the angle, spine and crest of the pubes. The *extensor longus digitorum pedis*, instead of having its four tendons divided into five, the outer tendon being inserted into the base of the metatarsal bone of the little toe, alongside of the *peroneus tertius* and also into the inner extensor tendon of the *extensor brevis digitorum pedis*. The *flexor brevis digitorum pedis*, divided into but three tendons, which were inserted into the three larger toes as described, the one for the lesser toe being absent.

Having finished what we have at present to say upon Myology, we pass to that portion of Angeiology, viz: The Arterial Circulation noticing some peculiarities in their distribution. In the division of the right common carotid artery, I found the internal branch to be three times the size of the external. The branches of the external were as follows: the Superior Thyroid normal; at the point of origin of the lingual a large trunk came off, which at half an inch divided into the lingual and facial, there was no mastoid artery, but the occipital and auricular were as usual. The ascending pharyngeal was also wanting, and the transverse facial came off in common with the division of the external into the temporal and internal maxillary, the temporal bifurcating as soon as it crossed the zygoma.

In a female subject dissected by one of the classes, the right carotid artery did not bifurcate until on a level with the inferior maxilla. The common carotid gave off the superior thyroid, and immediately on its bifurcation, gave off the facial and lingual by a common trunk. The left carotid did not bifurcate until it had passed the superior border of the thyroid cartilage nearly half an inch.

The right subclavian was of large size, its direction natural, but its distribution somewhat unusual. The vertebral, its first superior branch, was natural. The thyroid axis gave off but two branches, the inferior thyroid and superficialis cervicis, the supra scapular coming off from the axillary, and the posterior scapular from the third division of the subclavian passing between the fifth and sixth cervical nerves as they united in the formation of the brachial plexus. The profunda cervicis came off a little anterior to its natural position and instead of passing between the transverse processes of the seventh and eighth vertebra, passed between the sixth and seventh. This is an exception (as Cruveilhier in his work states that in the examination of forty cases with reference to this particular point, he never found the artery to pass in that direction.) At about four lines from its origin it gave off the superior intercostal, which wound around the under surface of the subclavian, passing beneath the vertebral vein. The internal mammary was of large size and was the only branch given off from its under surface.

Of the Axillary artery, the thoracico acromialis and superior thoracic came off by a common trunk, which is frequently the case. Of the Brachial Artery, the superior and inferior profunda came off side by side from the natural position of the superior forming almost an axis, each taking its proper direction and carrying out its regular distribution.

The Arterial distribution of the fore arm was normal, so also of the veins and nerves.

We may resume a consideration of some other departments of this subject, at a future opportunity.

## Figures and Tables

**Figure f1:**